# Validation of single‐step GBLUP genomic predictions from threshold models using the linear regression method: An application in chicken mortality

**DOI:** 10.1111/jbg.12507

**Published:** 2020-09-28

**Authors:** Matias Bermann, Andres Legarra, Mary Kate Hollifield, Yutaka Masuda, Daniela Lourenco, Ignacy Misztal

**Affiliations:** ^1^ Department of Animal and Dairy Science University of Georgia Athens GA USA; ^2^ UMR GenPhySE INRA Toulouse Castanet Tolosan France

**Keywords:** accuracy, binary trait, categorical trait, validation of genomic models

## Abstract

The objective of this study was to determine whether the linear regression (LR) method could be used to validate genomic threshold models. Statistics for the LR method were computed from estimated breeding values (EBVs) using the whole and truncated data sets with variances from the reference and validation populations. The method was tested using simulated and real chicken data sets. The simulated data set included 10 generations of 4,500 birds each; genotypes were available for the last three generations. Each animal was assigned a continuous trait, which was converted to a binary score assuming an incidence of failure of 7%. The real data set included the survival status of 186,596 broilers (mortality rate equal to 7.2%) and genotypes of 18,047 birds. Both data sets were analysed using best linear unbiased predictor (BLUP) or single‐step GBLUP (ssGBLUP). The whole data set included all phenotypes available, whereas in the partial data set, phenotypes of the most recent generation were removed. In the simulated data set, the accuracies based on the LR formulas were 0.45 for BLUP and 0.76 for ssGBLUP, whereas the correlations between true breeding values and EBVs (i.e. true accuracies) were 0.37 and 0.65, respectively. The gain in accuracy by adding genomic information was overestimated by 0.09 when using the LR method compared to the true increase in accuracy. However, when the estimated ratio between the additive variance computed based on pedigree only and on pedigree and genomic information was considered, the difference between true and estimated gain was <0.02. Accuracies of BLUP and ssGBLUP with the real data set were 0.41 and 0.47, respectively. This small improvement in accuracy when using ssGBLUP with the real data set was due to population structure and lower heritability. The LR method is a useful tool for estimating improvements in accuracy of EBVs due to the inclusion of genomic information when traditional validation methods as *k*‐fold validation and predictive ability are not applicable.

## 1 INTRODUCTION

Accuracy of estimation of genetic merit for livestock animals is a topic of concern in animal breeding. Usually, three measures are taken into account to evaluate the quality of genetic predictions: accuracy, bias and dispersion. Accuracy is defined as the correlation between true (TBV) and estimated breeding values (EBV). Accuracy values near one indicate a strong association between TBVs and EBVs, and values near zero indicate weak associations. Weak associations possibly imply that animals are poorly ranked, and selection decisions will be suboptimal. Bias refers to the difference between the average prediction and the average true value, which has a desirable value of zero. Biased predictions lead to incorrect comparisons between animals of different generations and inaccurate estimates of genetic trends (Henderson, Kempthorne, Searle, & Krosigk, 1959). Finally, dispersion can be understood as the predicted bias (slope of the regression of TBV on EBV) and has an expected value of one. If the dispersion is lower than one, the mean prediction for the test animals is biased downwards. On the other hand, if the dispersion is greater than one, the mean is biased upwards (Mäntysaari, Liu, & VanRaden, 2010).

With the availability of genomic information for livestock animals, validation techniques became widely used in breeding and genetics, especially to validate genomic models (Gianola & Schön, 2016). The data set is usually divided into a training and a validation set. The training set is used to fit a model, and the validation set is used to test the model using an objective function like mean square error (*MSE*) or correlation between predictions and observations. Several types of validations are available, and the choice depends on the properties of the data set. A validation technique applied to small data sets is the *k*‐fold validation which consists of randomly taking several data subsets and correlating the predictions obtained for the *k* fold when phenotypes for this fold are excluded (Saatchi et al., 2011). However, this technique does not take into account population structure. Hence, it is possible to have old animals in fold *k* and young animals in fold *k*‐1. In such cases, ancestors are predicted from progeny, which does not make sense in animal breeding (Thompson, 2001). For instance, if many animals are progeny of a small number of parents, then predictions will be close to that of parental averages and correlations between (G)EBVs among subsets may be very high. Additionally, these correlations are estimators of ratios of accuracies, as pointed out by Legarra and Reverter (2018). For dairy sires with a large number of progeny, validation is done by regressing daughter yield deviations (DYD) or deregressed proofs (DRP) obtained with the whole data set on EBV or GEBV for young bulls with no daughter information in the partial data set (VanRaden et al., 2009). The disadvantages of this type of validation are that DYD are difficult to compute, and best linear unbiased predictor (BLUP) is biased if genomic information is not accounted for by the evaluation model. A convenient measure of accuracy for animals with phenotypes is predictivity, which is defined as the correlation between GEBV or EBV computed with the partial data set and phenotypes adjusted for estimates of fixed effects (Legarra, Robert‐Granié, Manfredi, & Elsen, 2008). Predictivity can be used as an estimator of the accuracy when divided by the square root of heritability (Legarra & Reverter, 2018). However, this method may be difficult to apply to complex models (e.g. multiple random effects) and may lead to values greater than 1 if heritabilities are low and changing under selection. Properties of many methods for validation can be ascertained by analysing the decomposition of (G)EBV (Lourenco et al., 2015).

None of the above methods is applicable to categorical traits such as mortality, litter size, calving ease and disease resistance. Most of the statistical models used to handle categorical data, such as scoring models (Gianola & Foulley, 1983; Harville & Mee, 2006) or generalized linear models (Tempelman, 1997), link the categorical trait with an underlying continuous phenotype called liability. Hence, the observed phenotype can be interpreted as an expression of the liability. These models assign probabilities for each animal to express various possible phenotypes. Thus, a suitable validation approach for categorical data is to set cut‐off points for these probabilities and assign each animal a phenotype. However, these cut‐off points may be arbitrary and difficult to assign when probabilities are homogeneous among phenotypes. Another approach to validate statistical models for categorical data is to calculate receiver operating characteristic (ROC) curve (Park, Goo, & Jo, 2004). Although it is a useful approach to classify different models, the results cannot be interpreted as accuracies (Toghiani et al., 2017).

Recently, Legarra and Reverter (2018) developed the linear regressions (LR) method, which is a validation method based on LR and the R method (Reverter, Golden, Bourdon, & Brinks, 1994). They used the method of moments to derive estimators for bias, dispersion, accuracy and increase in accuracy when comparing “early” and “late” predictions. The method can be applied to virtually any statistical model provided that the assumptions of the model are fulfilled. The primary goal of this study was to apply and evaluate the method for a dichotomous trait with low incidence using simulated and commercial data sets. The secondary goal was to prove that the LR method yields consistent estimators of accuracy.

## 2 MATERIALS AND METHODS

### 2.1 LR method

In this study, we used estimators of population bias, dispersion and accuracy to evaluate genetic and genomic models. Let ***u*** and ***û*** be the vectors of TBVs and EBVs, respectively, then the (true) bias is defined as $\bar{u}-\bar{\hat{u}}$. The (true) dispersion, interpreted as the slope of the regression of ***u*** on ***û*** is equal to $\frac{cov(u,\hat{u})}{var(\hat{u})}$, where *cov* and *var* denote the sample covariance and variance, respectively. Finally, the accuracy is defined as the sample Pearson correlation coefficient between ***u*** and ***û***, which is equal to $\frac{cov(u,\hat{u})}{\sqrt{var\left(u\right)var\left(\hat{u}\right)}}$.

The LR method uses two data sets and a set of focal individuals. The *whole* data set contains all phenotypes, and the *partial* data set contains phenotypes up to a given date. The focal individuals are usually defined as a group of young animals of interest such as animals that might be selected at a given point in time given early (partial) information. The partial data set can be interpreted as the evaluation at the time of selection decisions, and the whole data set as a posteriori confirmation of the goodness of these selection decisions. Hereafter, subscript *w* will denote that an object comes from the whole data set and subscript *p* from the partial data set.

Legarra and Reverter (2018) suggested the following statistics for the three measurements in the LR method: (a) $\mu_{\mathrm{wp}}={\hat{u}}_{p}-{\hat{u}}_{w}$ for the bias, with an expected value of zero if the evaluation if unbiased; (b) $b_{w,p}=\frac{cov({\hat{u}}_{w},{\hat{u}}_{p})}{var({\hat{u}}_{p})}$ for the slope of the regression of the EBVs computed with the whole data set on the EBVs estimated with the partial data set, with an expectation value of one if the evaluation is neither underdispersed nor overdispersed; and (c) $\rho_{cov(w,p)}^{2}=\frac{cov({\hat{u}}_{w},{\hat{u}}_{p})}{\left(1+\bar{F}-2\bar{f}\right)\sigma_{u,\infty }^{2}}$ for the reliability (square of accuracy), where $\bar{F}$ is the average inbreeding coefficient, $2\bar{f}$ is the average relationship between individuals, and $\sigma_{u,\infty }^{2}$ is the genetic variance of the validation individuals. Additional estimators proposed by Legarra and Reverter (2018) that were also used in this research are: $\rho_{w,p}=cor\left({\hat{u}}_{w},{\hat{u}}_{p}\right)$, which estimates the ratio between the accuracies obtained with partial and whole data sets (i.e. ${\mathrm{acc}}_{p}/{\mathrm{acc}}_{w}$), and $\rho_{A,G}=cor\left({\hat{u}}_{A},{\hat{u}}_{G}\right)$, which is an estimator of the ratio between accuracies obtained by pedigree‐based and genomic‐based evaluations in the partial data set (i.e. ${\mathrm{acc}}_{A}/{\mathrm{acc}}_{G}$). The former can be expressed as the relative increase in accuracy by adding phenotypic information (${\mathrm{inc}}_{\mathrm{Phen}}=\rho_{w,p}^{-1}-1$), and the latter can be expressed as the increase in accuracy by adding genomic information to the partial data set (${\mathrm{inc}}_{G}=\rho_{A,G}^{-1}-1$).

### 2.2 Data simulation

A simulated data set, mimicking a chicken population, was generated with QMSim v 1.10 (Sargolzaei & Schenkel, 2009). The historical population began with 50,000 individuals and steadily decreased to 5,000 individuals after 1,000 generations. In this historical population, the generations were non‐overlapping, there was no selection and migration, and matings were random. A recent population was created by selecting 10 males and 4,500 females from the last generation of the historical population. Based on Wright's formula (Wright, 1931), the effective population size (Ne) was approximately 40, which agrees with the Ne in real chicken populations (Pocrnic, Lourenco, Masuda, & Misztal, 2016). Matings between males and females were random; hence, each sire was mated with 450 females on average. The recent population underwent selection for 10 generations. In every generation, each female had 1 offspring. From these offspring, males and females were selected based on high EBV to replace 25% of the sires and dams with lowest EBV. This process generated a pedigree with 49,510 birds.

Genotypes were simulated for from generations eight to 10 (*n* = 13,500). The simulated genome was composed of 38 chromosomes with length and number of QTL based on the Chicken QTL Database (www.animalgenome.org). Altogether, the number of simulated SNP and QTL was 41,989 and 9,505, respectively. The QTL accounted for all the genetic variation and their effects were simulated from a Gamma distribution (shape = 0.40), which resulted in QTL with small effects. All SNP and QTL had 0.5 allele frequencies in the first generation of the historical population. The recurrent mutation rate for QTL and SNP was assumed to be 2.5 x 10^–5^ per locus per generation.

Continuous phenotypes for generations 0 to 10 were calculated by combining a mean, the sum of QTL effects and the residual effect, for a heritability of 0.3. This continuous phenotype was transformed to a binary scale by setting the 7% lowest phenotypes in each generation to 1 (i.e. failure) and the remainder to 2 (i.e. success), according to survival rate in the field data. Animals recorded as dead were not able to have progeny. Birds from generation 10 (*n* = 4,500) were assigned to the validation group and had their phenotypes removed from the partial data set but included in the whole data set. The simulation was replicated five times.

### 2.3 Field data

A real data set with phenotypes for mortality on 186,596 broiler chickens was provided by Cobb‐Vantress Inc. The incidence of mortality was 7.2%, whereas the heritability on the underlying scale was 0.14 (the estimation based on data is described in the next section). Genotypes from the 60k SNP panel were available for 18,047 birds, and pedigree information was available for 188,935 birds. In these chicken line, there are several selection days. Mortality was recorded from hatch through the first selection day, although the exact date of mortality for each animal was not recorded; therefore, birds received 1 if dead and 2 if alive at the first day of selection. Consequently, all parents were recorded as alive. Also, animals were genotyped only if they were selected and, therefore, after the first selection date. Hence, all genotyped birds were recorded as alive. Quality control removed SNP with call rates lower than 0.9, minor allele frequencies lower than 0.05 and deviances from Hardy–Weinberg equilibrium >0.15 (Wiggans et al., 2009). Markers with unknown positions or located on sex chromosomes were also excluded from the analyses. After quality control, 39,102 SNP were kept for analysis. The set of focal individuals consisted of young animals from the last generation (*n* = 9,553) where 2,382 were genotyped. The incidence of mortality for the focal individuals was 10.0%.

### 2.4 Model and analyses

Threshold models (Gianola & Foulley, 1983) were fitted to the simulated (S) and real (R) data sets. Two models were fitted to each data set. The first model used only phenotypes and pedigree information (threshold model with pedigree: TM), whereas the second model utilized phenotypes, pedigree and genomic information (threshold model with pedigree and genomics: TMG). The acronyms for the two models were TMS and TMGS in the simulated data set and TMR and TMGR in the real data set. All models were single‐trait and included the generation of the animal as a fixed effect to account for environmental factors (all animals were kept in the same farm), and animal and residual as random effects. Variance components and (G)EBVs for each model were estimated using THRGIBBS1F90 (Tsuruta & Misztal, 2006). A uniform prior was assumed for fixed effects, whereas the additive genetic effect was assumed to be normally distributed with mean zero and variance $A\sigma_{u}^{2}$ when genomic information was not considered and $H\sigma_{u}^{2}$ when genomic information was considered, where **H** is the realized relationship matrix that combines pedigree‐ and genomic‐based relationships (Aguilar et al., 2010). For$\sigma_{u}^{2}$, a scaled inverted chi‐squared was used as prior distribution. For variance components, the Gibbs sampling process comprised 100,000 rounds and 1 every 10th sample was stored. After discarding the first 10,000 samples as burn‐in, posterior means were calculated. For (G)EBV, variance components were fixed to the posterior means and 10,000 samples were drawn. Variance components were calculated with and without genomic information. Posterior means of (G)EBV were used in the validation process. Under ssGBLUP, the inverse of **H** (**H** ^−1^) was used in the mixed‐model equations instead of the inverse of **A** (**A** ^−1^). The **H** ^−1^ is defined as:

$$H^{-1}=A^{-1}+\left[\begin{array}{cc} 0 & 0\\ 0 & G^{-1}-A_{22}^{-1} \end{array}\right]$$

where **G** ^−1^ is the inverse of the genomic relationship matrix (VanRaden, 2008), and $A_{22}^{-1}$ is the inverse of the pedigree‐based relationship matrix for genotyped animals.

To compute accuracies using the LR method, the genetic variance in the last generation, that is, $\sigma_{u,\infty }^{2}$, was obtained via Gibbs sampling using the approach proposed by Sorensen (2001) utilizing 10,000 samples. Subsequently, the statistics for the LR method were computed for each replicate of the simulated and real data sets. In addition, all statistics in the simulated data set were computed using TBV as a benchmark.

## 3 RESULTS

### 3.1 Analytical results

Appendix 1 shows the proof that $\rho_{Cov(w,p)}^{2}$ is a consistent estimator of the expected value of the reliability. This means that when the number of focal individuals is large enough, the expectation of $\rho_{Cov(w,p)}^{2}$ tends to be equal to the expectation of the reliability. In order for this to hold, ***u*** and ***û*** must be normally distributed, and the matrix of the *PEVs* for the validation animals in the partial data set should not tend to the null matrix. By the continuous mapping theorem on convergence in probability (Mann & Wald, 1943), $\sqrt{\rho_{Cov(w,p)}^{2}}$ is a consistent estimator of the expected accuracy value. Hence, $\sqrt{\rho_{Cov(w,p)}^{2}}$ can be interpreted as a consistent predictor of the accuracy.

Legarra and Reverter (2018) claimed that $\rho_{A,G}$ is a predictor of the ratio of accuracies ${\mathrm{acc}}_{A}/{\mathrm{acc}}_{G}$. In this paper, we suggest an improvement to this formula so that the ratio is better predicted. While the true genetic variance of the last generation is the same regardless of the model, the estimates may be different under genomic and non‐genomic models. Appendix 2 shows that in scenarios with complete pedigree, ${\left({\hat{\sigma }}_{A}^{2}/{\hat{\sigma }}_{G}^{2}\right)}^{-1/2}\rho_{A,G}$ using estimates of the respective genetic variances is a better predictor of the ratio of accuracies. Assuming that the ratio of variances is constant among generations, even under selection, the ordinary estimates of the variances can be used in any situation. This statistic leads to a consistent prediction of ${\mathrm{acc}}_{A}/{\mathrm{acc}}_{G}$.

### 3.2 Results based on simulated and real data

Table 1 shows estimated versus real values for bias, dispersion, accuracy and increase in accuracy by adding phenotypes to the simulated data set. The estimated bias was smaller and less variable than the true bias among different replicates, regardless of whether genomic information was used or not. In both cases, the true bias was approximately three times greater than the estimated value. When using either pedigree or pedigree and genomic information, the estimated and true dispersions were near one, although estimates were approximately 0.2 lower than the true values. However, when only pedigree information was used, the estimated dispersion was more variable. The true accuracy greatly increased (from 0.37 to 0.65) when genomic information was added, and this was reflected in the estimated accuracies by the LR method, although the estimates were greater than the true values. For pedigree‐based evaluation, the estimated accuracy was 0.08 greater than the true value, and this difference increased to 0.11 in the genomic‐based evaluation. The ratio ${\mathrm{acc}}_{p}/{\mathrm{acc}}_{w}$, which measures the increase in accuracy from the partial to the whole data set, was underestimated in both pedigree‐ and genomic‐based approaches. The difference between the estimated and true values in the pedigree‐based evaluation was 0.12 and with genomic information was 0.07. Consequently, the increase in accuracy (i.e. *Inc_phen_*) by adding phenotypes was overestimated by both approaches.

**TABLE 1:** Comparison between estimated and true values of bias, dispersion, accuracy, increase in accuracy by adding phenotypes and increase in accuracy by adding genomic information to the simulated data set with (TMGS) and without (TMS) genomic information; standard deviations reported in parenthesis

LR statistics	TMS		TMGS	
	Estimated^a^	True^b^	Estimated	True
Bias	0.07 (0.02)	0.28 (0.29)	0.07 (0.04)	0.26 (0.29)
Dispersion	0.96 (0.15)	1.17 (0.23)	0.95 (0.05)	1.12 (0.05)
Accuracy	0.45 (0.07)	0.37 (0.08)	0.76 (0.08)	0.65 (0.04)
*acc_p_*/*acc_w_* ^c^	0.83 (0.1)	0.95 (0.16)	0.91 (0.03)	0.98 (0.05)
*Inc_phen_* ^d^ (%)	20.4	5.2	9.8	2

^a^ The benchmark was the breeding value estimated using the whole data set.

^b^ The benchmark was the true breeding value.

^c^ Ratio between the accuracies obtained with partial and whole data set.

^d^ Increase in accuracy by adding phenotypes.

Table 2 shows the increase in accuracy by adding genomic information to the simulated data set. The estimation of the increase in accuracy resulting from adding genomic information was inflated compared to the true value when it was unadjusted by estimates of variance components in either pedigree or genomic models. When the variance ratio was included, this difference was significantly reduced.

**TABLE 2:** Increase in accuracy by adding genomic information with and without the variance ratio to the simulated data set

LR statistics	Estimated	True
*inc_G_* ^a^ without variance ratio^b^ (%)	85	75
*inc_G_* including variance ratio (%)	76	75

Note

^a^ Increase in accuracy by adding genomic information to the partial data set.

^b^ Estimated variance ratio $\left(\frac{{\hat{\sigma }}_{A}^{2}}{{\hat{\sigma }}_{G}^{2}}\right)$ = 0.9 (0.15).

Table 3 shows various statistics as well as estimated increases in accuracy by adding genomic information to the real data set. The estimated biases when using either only pedigree or pedigree and genomic information were close to zero. The dispersion was near 1 in both cases. This indicates that there is little over and under dispersion in the analysed data set. The estimated accuracy for the real data set was 0.41 when using only pedigree and 0.47 when using pedigree and genomic information. These numbers are low, which may be explained by the fact that dead animals are not genotyped (Garcia et al., 2018), the incidence of the trait and completeness of pedigree information. These three facts result together in a small difference between **A** ^−1^ and **H** ^−1^, hence in a small difference in estimated accuracies. The ratio ${\mathrm{acc}}_{p}/{\mathrm{acc}}_{w}$ was 0.03 greater for the genomic approach indicating that the increase in accuracy by adding phenotypes was similar to and without genomic information. The increase in accuracy by adding genomic information was 15% when the variance ratio was not considered. When the variance ratio was added, the increase in accuracy was 22%.

**TABLE 3:** Bias, dispersion, accuracy, increase in accuracy by adding phenotypes and increase in accuracy by adding genomic information to the real data set with pedigree information (TMR = real data set with pedigree information; TMGR = real data set with pedigree and genomic information)

LR statistics	TMR	TMGR
Bias	0.003	0.004
Dispersion	1.034	1.02
Accuracy	0.41	0.47
*acc_p_*/*acc_w_* ^a^	0.85	0.88
*Inc_phen_* ^b^ (%)	17	14
*inc_G_*without variance ratio^c^ (%)	15	
*inc_G_* including variance ratio (%)	22	

Note

Estimated variance ratio $\left(\frac{{\hat{\sigma }}_{A}^{2}}{{\hat{\sigma }}_{G}^{2}}\right)$ = 0.95.

^a^ Ratio between the accuracies obtained with partial and whole data sets.

^b^ Increase in accuracy by adding phenotypes.

^c^ Increase in accuracy by adding genomic information to the partial data set.

**FIGURE 1:**
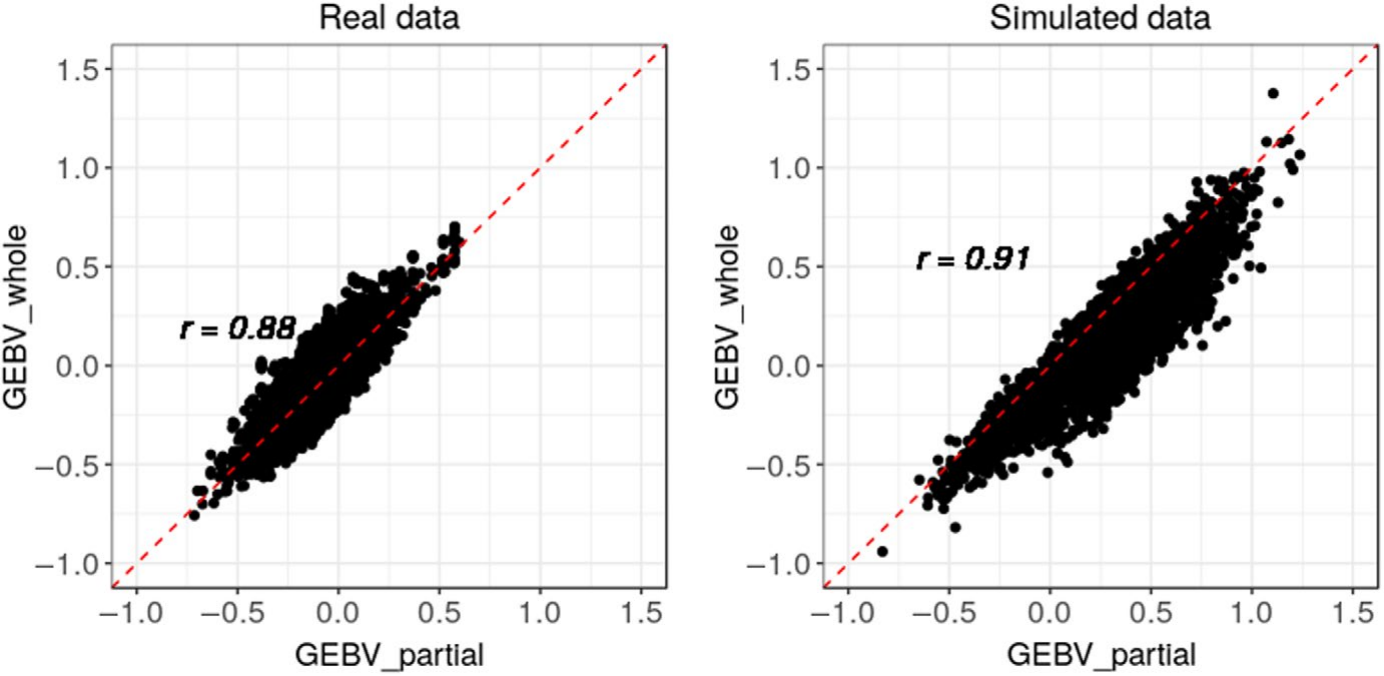
Scatter plot of the relationship between GEBVs for the partial and whole data sets from the real and simulated data sets. In each plot, r denotes the Pearson correlation coefficient

Figure 1 shows the distribution of the GEBVs from the partial data set against the GEBVs from the whole data set for both real and simulated data sets.

The graph for the simulated data set is slightly narrower (higher correlation), and it has visually few outliers.

## 4 DISCUSSION

In this study, we validated a threshold model for mortality in chickens using the LR method. Categorical traits can be handled with different models, like those from the Bayesian alphabet (Wang et al., 2013). LR method was derived based on mixed‐model assumptions; hence, it can be applied to any model that follows those assumptions.

Although the bias and dispersion from the simulated data set were slightly underestimated relative to the true values, the statistics based on EBV with partial and whole data sets showed that pedigree and pedigree and genomic models were unbiased and had little over and under dispersion. Given that differences between estimated and true values were observed, it seems the LR statistics pointed in the right direction, but with different magnitudes.

Accuracies in the simulated data set were slightly overestimated relative to the true values both with and without genomic information. The LR method uses the frequentist distribution of predictands and predictors assuming that phenotypes have a continuous distribution. If this assumption is violated, the (G)EBVs of the focal individuals may not be normally distributed. In such case, the consistency of $\rho_{Cov(w,p)}^{2}$ does not hold. Another source of variability for the prediction of accuracy with $\rho_{Cov(w,p)}^{2}$ is the adjusted additive variance for set of focal individuals. Because $\rho_{Cov(w,p)}^{2}$ is inversely proportional to $\sigma_{u,\infty }^{2}$, we can infer that the estimated adjusted variance is too small. In fact, the estimated accuracy was 0.45 with $\sigma_{u,\infty }^{2}$ and 0.41 when using $\sigma_{u}^{2}$ in a pedigree‐based model. In our case, when no selection was assumed, the estimation of accuracy was closer to the true value (0.37). Since performance of LR method depends on model specification (Macedo, Reverter, & Legarra, 2020), model misspecification and population structure may also explain the obtained results. In their results, the consistency of $\rho_{Cov(w,p)}^{2}$ can be observed when the model is correctly specified.

The gain in accuracy by adding genomic information is a topic of concern for genetic evaluations. Denoting a pedigree‐based evaluation with the subscript *A* and a genomic‐based evaluation with the subscript *G*, Legarra and Reverter (2018) claimed that the correlation between *ρ_A,G_* is an estimator of the ratio of the accuracies of the two models. In this paper, we propose to improve this estimator by accounting for differences in estimated values of variance components based on pedigree and pedigree plus genomic information, that is, ${\left({\hat{\sigma }}_{A}^{2}/{\hat{\sigma }}_{G}^{2}\right)}^{-1/2}\rho_{A,G}$. Usually, $\sigma_{A}^{2}$ and $\sigma_{G}^{2}$ are assumed to be equal. This means that the pedigree‐based relationship matrix (**A**) in BLUP and the realized relationship matrix (**H**) in the ssGBLUP approach are deemed to use the same variance components. However, their estimators can be different. The estimate of the variance using a pedigree‐based approach was 0.21 compared to 0.23 when genomic information was used in the simulated data set. Conversely, these values were 0.16 and 0.13 in the real data set, respectively. This shows that the assumption of equality of estimated variances is reasonable, and its impact on the estimation of breeding values is limited. Despite these small differences, the performance of the estimation of the ratio ${\mathrm{acc}}_{A}/{\mathrm{acc}}_{G}$ was improved by adding the estimators of the variance in the statistic. The true value of ${\mathrm{acc}}_{A}/{\mathrm{acc}}_{G}$ was approximately 0.57 in the simulated data set. This implies that the increase in accuracy due to addition of genomic information was equal to 75%. The estimated value of ${\mathrm{acc}}_{A}/{\mathrm{acc}}_{G}$ was equal to 0.54 which represents an increase in accuracy of 85%. The estimator ${\left({\hat{\sigma }}_{A}^{2}/{\hat{\sigma }}_{G}^{2}\right)}^{-1/2}\rho_{A,G}$ was approximately equal to 0.56. This value denotes an increase in accuracy equal to 76%, indicating that adding the reciprocal of the square root of the variance ratio resulted in a more accurate prediction of ${\mathrm{acc}}_{A}/{\mathrm{acc}}_{G}$. Consequently, the prediction of the increase in accuracy by adding genomic information was also improved from 85% to 76%, while the true increase of accuracy was 75%.

The statistics from the real data set showed no signs of bias and dispersion. The increase in accuracy due to the utilization of genotypes was small. This can be explained by a lower heritability in the real data set than in the simulated data set and no genotyping of dead animals.

Zhang et al. (2018) estimated average accuracies for mortality and disorder traits in chickens using threshold models to be 0.47 without genomic information and 0.54 with genomic information; this resulted in an increase in accuracy of 0.07 compared to 0.06 in this study. Their validation procedure was based on Ramirez‐Valverde, Misztal, and Bertrand (2001), who proposed to randomly split the data set into halves, using one half for prediction and the other half for validation. In their research, the correlation between the predicted breeding values of the two subsets was the selected loss function (Gianola & Schön, 2016). As mentioned by Thompson (2001), this method of splitting a data set into halves can lead to problems. Also, the application of data splitting is more complex than the LR method especially for large data sets.

Legarra and Reverter (2018) emphasized that correlation between predictions obtained using whole and partial data sets is not a measure of accuracy, but an estimator of the ratio between accuracies. When the ratio of accuracies is equal to 0.55, the corresponding increase in accuracy from partial to whole data sets is 81%. Some studies have used this correlation as a pure measure of accuracy without emphasizing its proper meaning (Legarra & Reverter, 2018). In our research, the estimated ratio of accuracies in the simulated data set was 0.83 for the pedigree‐based evaluation and 0.91 for the genomic‐based evaluation. This indicates an increase in accuracy of 20% for the pedigree‐based evaluation and 9.8% for the genomic‐based evaluation when using the whole data set. Because the focal individuals in our study included only animals from the last generation (10% of the data set), the increase in accuracy by adding phenotypes for these animals is likely to be smaller than that of Zhang et al. (2018).

## 5 CONCLUSION

The LR method is a useful tool for estimating the magnitude of bias, dispersion and accuracy for threshold models. This method is applicable to any model, single‐step procedures, and it is easy to run. More accurate estimates are possible when estimates of variances in the final generation are known but at a cost of potentially expensive computing. When applied to analysis of mortality in broiler chickens using a threshold model, the LR method showed a moderate improvement in accuracy due to the use of genomic information.

## CONFLICT OF INTEREST

The authors declare that they do not have any conflict of interest.

## Data Availability

The data belongs to Cobb‐Vantress and, therefore, cannot be shared.

## Appendix 1

### Proof of consistent predictor of the reliability

Here, we show that $\rho_{Cov(w,p)}^{2}$, defined as $\frac{cov({\hat{u}}_{w},{\hat{u}}_{p})}{\left(1+\bar{F}-2\bar{f}\right)\sigma_{u,\infty }^{2}}$ in Legarra and Reverter (2018), is a consistent predictor of the reliability. We assume that random effects are normally distributed, their means are homogeneous within each effect, and the covariance matrix for the joint distribution of ***u***, ***û*** *_w_* and ***û*** *_p_* is

(1) $$Var\left[\begin{array}{c} u\\ {\hat{u}}_{p}\\ {\hat{u}}_{w} \end{array}\right]=\left[\begin{array}{c} \begin{array}{ccc} G & G-C_{p}^{\mathrm{uu}} & G-C_{w}^{\mathrm{uu}}\\ & G-C_{p}^{\mathrm{uu}} & G-C_{p}^{\mathrm{uu}}\\ symm & & G-C_{w}^{\mathrm{uu}} \end{array} \end{array}\right]$$

where **G** is the relationship matrix among individuals and **C** *^uu^* is the block corresponding to random effects of the inverse of the mixed‐model equations (Henderson, 1982).

As a consistent estimator, $\rho_{Cov(w,p)}^{2}$ must fulfil the two following conditions:

1. The expected value of $\rho_{Cov(w,p)}^{2}$ must converge to the reliability; and
2. The variance of $\rho_{Cov(w,p)}^{2}$ must tend to zero when the number of focal individuals tends to infinity.

Let *n* be the number of focal individuals and *N* be the number of animals in the whole evaluation set. Let *rel* = *X*/*Y* be the reliability of the focal individuals, where $X=\left(u^{\prime }S\hat{u}\right)\left(u^{\prime }S\hat{u}\right)$ and $Y=\left(u^{\prime }Su\right)\left({\hat{u}}^{\prime }S\hat{u}\right)$. Then, as n→N→∞:

(2) $$rel\overset{p}{\to }=\frac{tr\left[S\left(G-C_{p}^{\mathrm{uu}}\right)\right]}{tr\left(\mathrm{SG}\right)}=1-\frac{\bar{\mathrm{PEV}}-\bar{\mathrm{PEC}}}{\left(1+\bar{F}-2\bar{f}\right)\sigma_{u,\infty }^{2}}$$

where **S** = **I** *_n_* −*n* ^−1^ **J** *_n_* and $\overset{p}{\to }$ denotes convergence in probability.

To prove (2), note that by the Law of Large Numbers (Gnedenko, 1978 Ch. 6) the sample variances of u and $\hat{u}$, and the sample covariance between u and $\hat{u}$ converge in probability, as *n* tends to infinity, to their expectations. Hence:

(3) $$\begin{array}{c} {\left(n-1\right)}^{-1}u^{\prime }S\hat{u}\overset{p}{\to }E\left[{\left(n-1\right)}^{-1}u^{\prime }S\hat{u}\right]={\left(n-1\right)}^{-1}tr\left[S\left(G-C_{p}^{\mathrm{uu}}\right)\right]\\ {\left(n-1\right)}^{-1}{\hat{u}}^{\prime }S\hat{u}\overset{p}{\to }E\left[{\left(n-1\right)}^{-1}{\hat{u}}^{\prime }S\hat{u}\right]={\left(n-1\right)}^{-1}tr\left[S\left(G-C_{p}^{\mathrm{uu}}\right)\right]\\ {\left(n-1\right)}^{-1}u^{\prime }Su\overset{p}{\to }E\left[{\left(n-1\right)}^{-1}u^{\prime }Su\right]={\left(n-1\right)}^{-1}tr\left[\mathrm{SG}\right] \end{array}$$

Then, using the properties of the limit, the sample correlation coefficient between u and $\hat{u}$ (i.e. the accuracy) converges in probability, to its expected value:

(4) $$\frac{u^{\prime }S\hat{u}}{\sqrt{u^{\prime }Su\;{\hat{u}}^{\prime }S\hat{u}}}\overset{p}{\to }\frac{tr\left[S\left(G-C_{p}^{\mathrm{uu}}\right)\right]}{\sqrt{tr\left[\mathrm{SG}\right]tr\left[S\left(G-C_{p}^{\mathrm{uu}}\right)\right]}}=\sqrt{\frac{tr\left[S\left(G-C_{p}^{\mathrm{uu}}\right)\right]}{tr\left[\mathrm{SG}\right]}}=\sqrt{1-\frac{\bar{\mathrm{PEV}}-\bar{\mathrm{PEC}}}{\left(1+\bar{F}-2\bar{f}\right)\sigma_{u,\infty }^{2}}}.$$

Since the square root is continuous and the domain of the accuracy is positive, the result in (2) follows from (4) by applying the continuous mapping theorem on convergence in probability (Mann & Wald, 1943). Noting that the expected value of $\rho_{Cov(w,p)}^{2}$ is asymptotically equal to *rel* with probability equal to one, the first condition is fulfilled.

To prove the second condition, first take the variance of $\rho_{Cov(w,p)}^{2}$:

(5) $$Var\left(\rho_{Cov\left(w,p\right)}^{2}\right)=Var\left(\frac{cov\left({\hat{u}}_{p},{\hat{u}}_{w}\right)}{\left(1+\bar{F}-2\bar{f}\right)\sigma_{u,\infty }^{2}}\right)=\frac{Var\left(n^{-1}{\hat{u}}_{p}^{\prime }S{\hat{u}}_{w}\right)}{{\left(\left(1+\bar{F}-2\bar{f}\right)\sigma_{u,\infty }^{2}\right)}^{2}}.$$

Then, as in (3), the sample covariance between ${\hat{u}}_{p}$ and ${\hat{u}}_{w}$ $\left({\left(n-1\right)}^{-1}{\hat{u}}_{p}^{\prime }S{\hat{u}}_{w}\right)$ converges in probability to its expectation, which is a constant. Since $n^{-1}{\hat{u}}_{p}^{\prime }S{\hat{u}}_{w}$ and ${\left(n-1\right)}^{-1}{\hat{u}}_{p}^{\prime }S{\hat{u}}_{w}$ converge to the same constant, both variances tend to zero with probability one, and as n→N→∞ we have that:

(6) $$Var\left(\rho_{Cov\left(w,p\right)}^{2}\right)=Var\left(\frac{\text{cov}\left({\hat{u}}_{p},{\hat{u}}_{w}\right)}{\left(1+\bar{F}-2\bar{f}\right)\sigma_{u,\infty }^{2}}\right)=\frac{Var\left(n^{-1}{\hat{u}}_{p}^{\prime }S{\hat{u}}_{w}\right)}{{\left(\left(1+\bar{F}-2\bar{f}\right)\sigma_{u,\infty }^{2}\right)}^{2}}\overset{p}{\to }0.$$

Since the expected value of $\rho_{Cov(w,p)}^{2}$ is is asymptotically equal to the reliability and the variance of $\rho_{Cov(w,p)}^{2}$ tends to zero with large *n*, $\rho_{Cov(w,p)}^{2}$ is a consistent predictor of the reliability.

## Appendix 2

### Impact of variance ratios on accuracies

This appendix shows that ${\left({\hat{\sigma }}_{A}^{2}/{\hat{\sigma }}_{G}^{2}\right)}^{-1/2}\rho_{A,G}$ is an appropriate predictor of the ratio of accuracies.

Let $Var\left(u_{G}\right)=K,Var\left({\hat{u}}_{G}\right)=K-C_{G}^{\mathrm{uu}}$ and $Cov\left(u_{G},{\hat{u}}_{G}\right)=Var\left({\hat{u}}_{G}\right)$, where **K** is the relationship matrix based on genomic information times a genetic variance and assume that $Cov\left({\hat{u}}_{G},{\hat{u}}_{A}\right)=Var\left({\hat{u}}_{A}\right)$ (Legarra & Vitezica, 2015). Following the same steps as in Appendix 1, it can be shown that:

(7) $$\rho_{A,G}^{2}=\frac{\left({\hat{u}}_{A}^{\prime }S{\hat{u}}_{G}\right)\left({\hat{u}}_{G}^{\prime }S{\hat{u}}_{G}\right)}{\left({\hat{u}}_{A}^{\prime }S{\hat{u}}_{A}\right)\left({\hat{u}}_{G}^{\prime }S{\hat{u}}_{G}\right)}\overset{p}{\to }\frac{tr\left[S\left(G-C_{p}^{\mathrm{uu}}\right)\right]}{tr\left(S\left(K-C_{G}^{\mathrm{uu}}\right)\right)}=\frac{\left(1+\bar{F}-2\bar{f}\right)\sigma_{A}^{2}}{\left(1+\bar{K}-2\bar{k}\right)\sigma_{G}^{2}}\frac{rel_{A}}{rel_{G}}.$$

When pedigree information is complete, $\left(1+\overset{-}{F}-2\overset{-}{f}\right)≅\left(1+\overset{-}{K}-2\overset{-}{k}\right)$, and (7) simplifies to:

(8) $$\rho_{A,G}^{2}\overset{p}{\to }\frac{tr\left[S\left(G-C_{p}^{\mathrm{uu}}\right)\right]}{tr\left(S\left(K-C_{G}^{\mathrm{uu}}\right)\right)}=\frac{\sigma_{A}^{2}}{\sigma_{G}^{2}}\frac{{\mathrm{rel}}_{A}}{{\mathrm{rel}}_{G}}.$$

Therefore:

(9) $${\left(\frac{\sigma_{A}^{2}}{\sigma_{G}^{2}}\right)}^{-1}\rho_{A,G}^{2}\overset{p}{\to }\frac{{\mathrm{rel}}_{A}}{{\mathrm{rel}}_{G}}.$$

The variance of the left‐hand side of (9) tends to zero as n→N→∞ (Hotelling, 1953). Therefore,${\left(\frac{{\hat{\sigma }}_{A}^{2}}{{\hat{\sigma }}_{G}^{2}}\right)}^{-1}\rho_{A,G}^{2}$ is a consistent predictor of the ratio of reliabilities and ${\left(\frac{{\hat{\sigma }}_{A}^{2}}{{\hat{\sigma }}_{G}^{2}}\right)}^{-1\;/\;2}\rho_{A,G}$ is a consistent predictor of the ratio of accuracies.
